# Inadvertent posterior intercostal artery puncture and haemorrhage after ultrasound-guided thoracic paravertebral block: a case report

**DOI:** 10.1186/s12871-018-0667-5

**Published:** 2018-12-21

**Authors:** Linlin Song, Yin Zhou, Da Huang

**Affiliations:** Department of Aanesthesiology, Peking University First Hospital, No. 15 Xishiku Street, Xicheng District, Beijing, 100034 China

**Keywords:** Paravertebral haemorrhage, Posterior intercostal artery, Thoracic paravertebral block, Ultrasound-guided

## Abstract

**Background:**

This report describes one case of paravertebral haemorrhage after ultrasound-guided thoracic paravertebral block (TPVB) that may have been attributed to the inadvertent puncture of the posterior intercostal artery (PIA). This complication has never been reported in ultrasound-guided TPVB. Strategies to prevent this potentially serious complication are discussed.

**Case presentation:**

A 52-year-old male underwent a video-assisted upper lobectomy. TPVB was performed under the guidance of ultrasound using the out-of-plane parasagittal approach. The transducer was placed 2.5 cm lateral to the midline area in a sagittal orientation. A needle was inserted at the lateral side of the transducer and advanced toward the T4 paravertebral space. During the final attempt, the needle tip was visualised in the middle area of the paravertebral space. Anterior displacement of the pleura was visualised upon injection of the saline. Aspiration of red blood was unfortunately identified. The block in this T4 level was discontinued. The patient was haemodynamically stable. When the chest cavity was entered, a bulging column-shaped haematoma was noted in the left paravertebral space extending from T1 to T12 with concomitant spread into the left T4–5 intercostal space. A postoperative neurological examination revealed intact sensory function in the T4 dermatome bilaterally. The patient fully recovered with no neurological sequelae.

**Conclusions:**

Ultrasound-guided TPVB still bears the potential risk of inadvertent PIA injury. We recommend colour Doppler imaging to identify PIA prior to the TPVB**.**

## Background

Thoracic paravertebral block (TPVB) is a technique in which local anaesthetics are injected into the thoracic paravertebral space (PVS), which is lateral to the intervertebral foramina, resulting in ipsilateral somatic and sympathetic nerve blockade that spreads from the site of injection across multiple contiguous dermatomes. TPVB has been successfully used in both adults and children to provide anaesthesia and analgaesia for multiple thoracic and abdominal procedures. However, TPVB is an advanced regional block. Care must be taken to prevent pneumothorax, local anaesthetic systemic toxicity, vascular injury and other potentially serious complications. Over the past ten years, by utilising ultrasound technology, the approaching needle can be guided by real-time imaging, thereby improving efficacy and safety for TPVB. Some reports have indicated high success rates and very few complications associated with an in-plane transverse ultrasound-guided TPVB approach [1]. Here, we describe a case of paravertebral haemorrhage after ultrasound-guided TPVB using the out-of-line parasagittal approach, which may be attributed to the inadvertent puncture of the posterior intercostal artery (PIA). We present this case of paravertebral haematoma for its value in illustrating the real risk of vascular injury complication even with the guidance of ultrasound. Furthermore, this case reminds us of an important step prior to the performance of TPVB-colour Doppler imaging to identify PIA, which could help prevent major vessel injury. So far, this step has not drawn much attention by TPVB practitioners. We obtained written informed consent from the patient for procedure and publication.

## Case presentation

An otherwise healthy 52-year-old male (172 cm tall and weighing 74 kg) was scheduled to undergo video-assisted upper lobectomy for left lung cancer.

Thoracic paravertebral block (TPVB) was planned using an Esaote ultrasound machine ((MyLab™Alpha, Esaote, Italy) and a low-frequency curvilinear transducer. We chose to perform the TPVB using the out-of-plane parasagittal approach since that is our department’s custom. After placing the patient in the right lateral position, the transducer was placed 2.5 cm lateral to the midline in a sagittal orientation, slightly oblique toward lateral [2]. Paravertebral space (PVS) between the T4 and T5 transverse processes was detected. This location was between the superior costotransverse ligament and the pleura. A 5-cm 22 G needle (Stimplex®D, B. Braun, Germany) was inserted at the lateral side of the transducer slightly toward medial. During the advancement, the needle tip was not visualised on the ultrasound screen. Only tissue displacement could be seen. Several attempts were performed. At the last attempt, the needle tip was visualised just below the superior costotransverse ligament in the middle of PVS. After a further advancing the needle, anterior displacement of the pleura in the centre of T4–5 PVS was visualised upon injection of the saline. Just before the local anaesthetics were available to be administered, aspiration of red blood was identified. The TPVB in this T4 level was discontinued. Again, we detected the T6 paravertebral level, the technique was the same as that in the T4 level. This time, the entire procedure was uneventful. Appropriate needle tip location was confirmed by displacement of pleura with widening of the intercostal space after injection of the saline. Aspiration through the needle was negative. Fifteen millilitres of 0.4% ropivacaine was injected. During the whole procedure the patient did not have any discomfort, pain or sign of pleural irritation. He was haemodynamically stable.

**Fig. 1:**
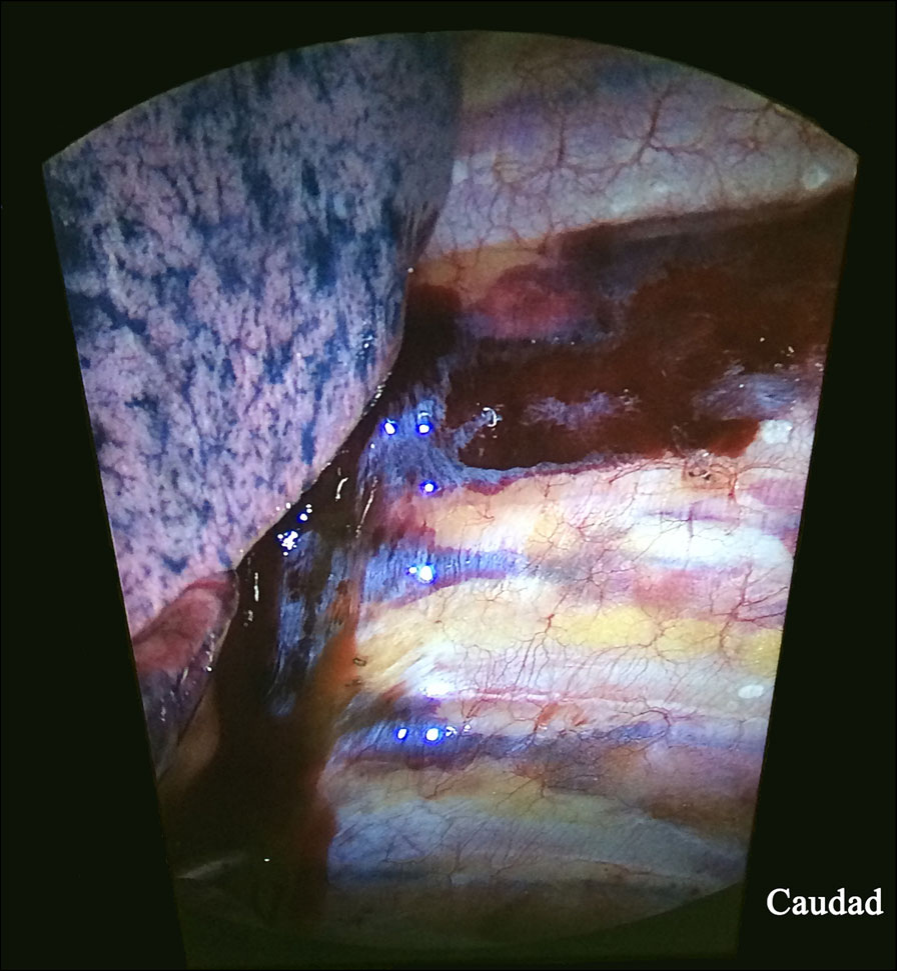
Paravertebral haematoma after inadvertent posterior intercostal artery puncture during the thoracic paravertebral block

When the chest cavity was entered, the surgeon found that in the left PVS underlying the pleura, there was a bulging, column-shaped haematoma extending from T1 to T12 with concomitant spread into the left T4–5 intercostal space to the post-axillary line (Fig. 1). No injury to the lung tissue was identified. The haematoma was left untouched. One gram of tranexamic acid was infused over 15 min. The operation was carried out as according to routine protocol and was uneventful.

Following the operation, the patient was started on an intravenous patient-controlled analgaesia (PCA) with sufentanil. On postoperative day 1, the patient complained of severe dynamic pain of 8/10 on a numeric rating score in the nipple area that was not alleviated by the intravenous PCA. Rescue analgaesia was given. A neurologic examination revealed intact sensory function in the T4 dermatome bilaterally and diminished sensation in the left T5-T7 dermatomes. The patient made a full recovery with no neurological sequelae and was discharged one week later.

## Discussion and conclusions

Given the extensive haematoma identified, we postulated that the posterior intercostal artery (PIA) was inadvertently punctured. In our case, no neurological sequelae or devastating complications were identified, which may be partly attributed to the normal haemostatic profile of this patient. Given a patient with coagulopathy, excessive blood loss due to PIA injury may lead to haemodynamic instability, compression of the lung and even spinal haematomas that are difficult to resolve surgically or radiologically. In the era of performing the TPVB without ultrasound, inadvertent vascular punctures associated with positive aspiration of blood occurred in 6.8% of the adult cases. Incidence of haematoma at site of injection is around 1.9–3.1% [3]. In consideration of the prevalence of small vessels that are normally present in adipose tissue, we speculated that minor vascular puncture may not be an infrequent incident during the performance of ultrasound-guided paravertebral block, not to mention the intercostal vessels. Most of them may be not symptomatic and are left unrecognised by anaesthetists, especially in operations in which the thorax is not opened. Although the haematoma is limited in magnitude, the regional anaesthesiologist should assure optimisation of the haemostatic status prior to the performance of TPVB. It is suggested that coagulopathy, bleeding abnormality, and therapeutic anticoagulation are relative contraindications of TPVB. Heightened vigilance is important to identify any new neurological dysfunction for these patient groups.

The TPVS is filled with adipose tissue that contains the intercostal nerve, artery and vein, and the sympathetic trunk. PIAs originate from the descending thoracic aorta, one or two segmental levels below their feeding levels. Each segmental artery then runs upward to reach the corresponding intercostal space. A study used the T4 to T8 intercostal space for measurements [4]. In the posterior paravertebral region, the PIA lies approximately in the middle of the intercostal space. Moving laterally, the PIA deviates superiorly and is located approximately one-third of the way from the superior rib at the angle of the rib. These anatomical studies provide some hints for the performance of the TPVB. For the sagittal technique at the transverse process level, just as that adopted in our case, keeping the needle tip away from the middle area in the targeted paravertebral region on the ultrasound screen should be sought, with an aim to avoid the centrally-situated PIA.

Unfortunately, there is a substantial amount of variability in PIA’s position, with less variability seen more laterally. Helm et al. stated that from T7 to T11 at the tip of the transverse process, only 17% of arteries were completely shielded by the superior rib, compared with 97% at 6 cm lateral to the midline [5]. In older patients and in higher rib spaces the arterial position is more variable. Vessel shape could be either straight or extremely tortuous. Even if the centre axis of the artery is more superiorly positioned within the intercostal space, the trajectory of the artery may travel through the relatively inferior portion of the intercostal space as tortuosity increases [6].

**Fig. 2:**
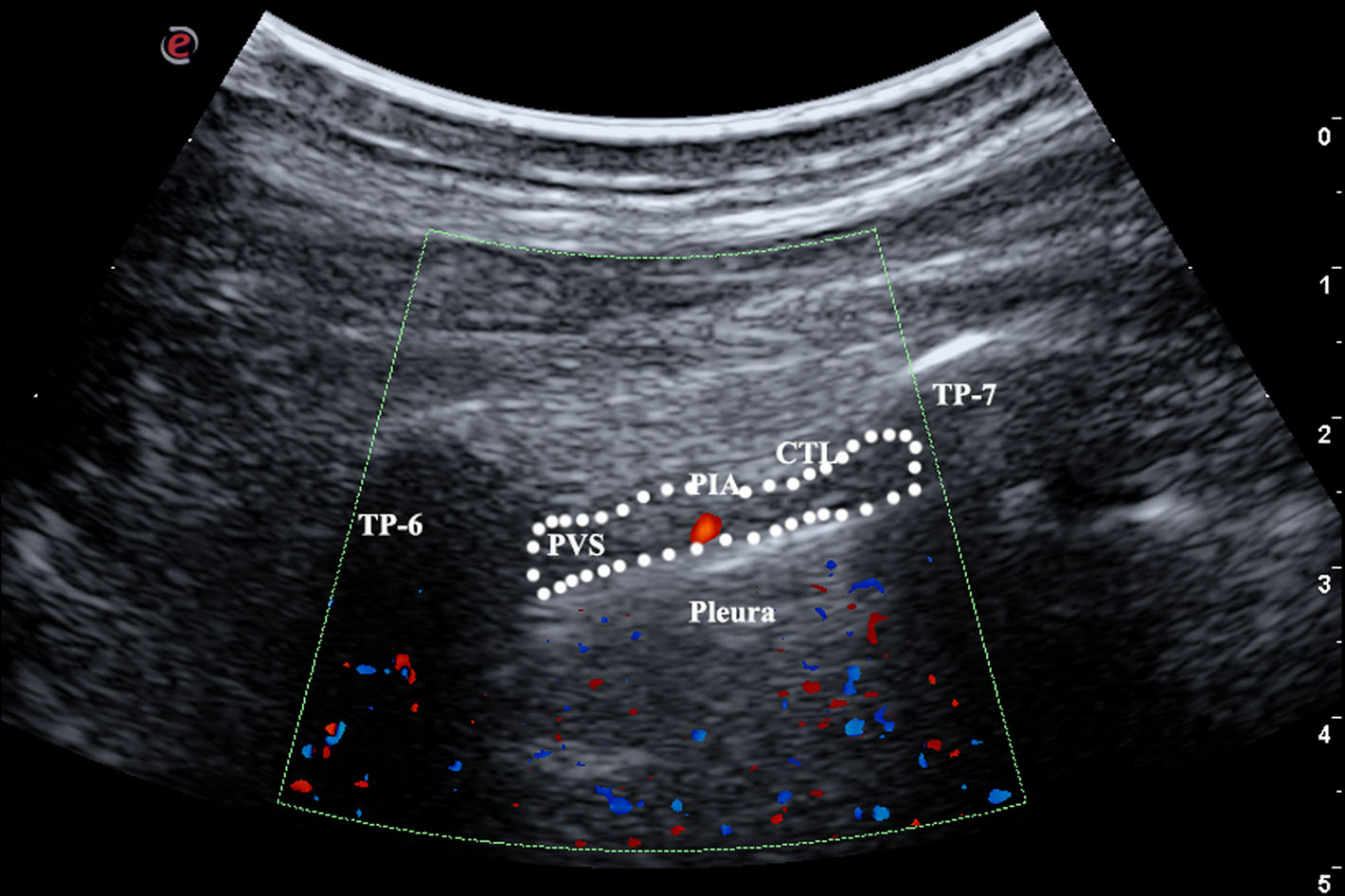
Parasagittal sonogram of the T6 thoracic paravertebral region 2.5 cm lateral to the midline area. *CTL* costo-transverse ligament, *PIA* posterior intercostal artery; *PVS* paravertebral space, *TP* transverse process

Although we are unable to accurately “predict” the location of PIA, colour Doppler imaging allows direct visualisation of PIA [7] (Fig. 2). Routine imaging PIA before performing the TPVB could decrease the risk of PIA puncture. We strongly recommend that this step should be followed by all TPVB performers. However, the absence of ultrasound detection of the PIA cannot currently be taken as evidence of a safe site for intervention within the 6-cm lateral to midline area. In some of the patients, it can be difficult to image PIA due to its small size and the non-perpendicular position of the ultrasound probe to it. Finally, a meticulous technique involving frequent aspiration should be keep in mind all the time.

It is possible that the in-plane puncture approach could be safer than the out-of-plane one in this case. However, an anaesthesiologist’s experience and preference are major determinants of the in-plane and an out-of-plane approach [8]. In the out-of-plane approach, the needle tip cannot be constantly monitored, but the shortest possible needle trajectory can be achieved.With the in-plane approach, the needle pathway could be traced continuously. Nevertheless, an optimal PVS image is generally difficult to acquire while the needle is advanced in the immediate proximity to both pleura and intervertebral foramina. In the case of visible needle tip in the PVS, the risk of vascular injury is empirically comparable between the in-plane and out-of-plane approaches. Regardless of the approach chosen, colour Doppler to identify PIA before the puncture may help decrease the risk of major vascular injury.

## Conclusions

Ultrasound-guided TPVB still bears the potential risk of inadvertent PIA injury and haematoma formation. We recommend colour Doppler imaging to identify PIA prior to the performance of TPVB.

## Abbreviations

PIA: Posterior intercostal artery
PVS: Paravertebral space
TPVB: Thoracic paravertebral block
